# Developmental assets and positive youth development in Brazilian university students

**DOI:** 10.3389/fpsyg.2022.977507

**Published:** 2022-10-04

**Authors:** Maurício Coelho de Jesus, Luciana Dutra-Thomé, Anderson Siqueira Pereira

**Affiliations:** ^1^Instituto de Psicologia, Federal University of Bahia, El Salvador, Bahia, Brazil; ^2^Wainer Psicologia Cognitiva, Porto Alegre, Brazil

**Keywords:** positive youth development, social connectedness, mental health, stressful events, university students

## Abstract

The Positive Youth Development (PYD) describes an intersection between young people and their context, emphasizing characteristics of a healthy development. The PYD’s 5Cs occur when there is an alignment between healthy individual characteristics and contextual resources. This study investigated the PYD’s 5Cs associations with the perception of social connections (family, community, academic), mental health, and stressful events. The sample was composed of 495 Brazilian College students aged between 18 and 33 years, who answered a survey with 59 questions about reflexive, constructive, and healthy behaviors. Descriptive, correlational, and regression analysis through structural equation modeling were conducted. The results focused on the role of family, community and educational institution in the PYD promotion. These results highlight the relevant contributions of social support in the construction of protective strategies of coping with stressful events and in the promotion of health behaviors and well-being, particularly in the university context.

## Introduction

Positive youth development (PYD) is a theoretical perspective regarding the potential that supportive and mutually beneficial relationships among young people and the context play in their development (Lerner, 2005). Change is both a consequence and a cause of developmental processes (Bronfenbrenner, 1998). In PYD, the Bioecological Model of Development (Bronfenbrenner, 1998) helps to reveal that the trajectory of young people during development is not fixed and can be influenced by factors in their homes, schools, work and communities (Lerner and Lerner, 2011). Change will emerge through the individual differences of each young person and the environment in which they are inserted. Not every young person experiences developmental transitions in the same way, at the same speed or with equal results (Lerner, 2005). In addition to considering the construction of youth identity and the person’s effective participation in the civic community (Crocetti et al., 2014), PYD considers the family, the community and the educational institution in the provision, maintenance and potentialization of healthy individual and contextual resources in promoting positive development (Copetti and Krebs, 2004; Bowers et al., 2010).

For measuring PYD, Lerner et al. (2005) proposed and tested a model through five latent constructs, each representing one of the Cs: Competence (intellectual and behavioral skills); Confidence (sense of self-efficacy and self-validation); Character (integrity, sense of right and wrong, and moral values); Connection (positive ties with people and institutions); and Caring (empathy and sympathy; Lerner, 2005). Young people who live with the five Cs incorporated into their lives will develop a sixth C as a result of this integration (Lerner, 2005), named as Contribution to themselves, to the family, to the community and to the institutions of civil society Based on the PYD model, the present study investigated the developmental context of young university students through the analysis of the associations between the five Cs of PYD and three variables, two classified as external assets, namely (1) connections of young people with the family, the educational institution and the community, and (2) exposure to stressful events; and one variable classified as an internal asset, namely (3) positive mental health.

In order to comprehend the cultural and social diversity experienced by young people, researchers have been engaged in the production and application of aspects of PYD in different contexts around the world (Adams et al., 2019; Dutra-Thomé et al., 2019; Kozina et al., 2019; Tirrell et al., 2019; Wiium et al., 2019). These studies have been based on topics such as spirituality and hope in young people, risk and protective factors among young adults, and academic performance, as well as contextual factors in each country.

In Brazil, some studies on PYD showed that young people from São Paulo did not have a favorable perception of acceptance, support and valorization in the relationships they had with the community and other groups (e.g., family, institutions). They also felt a lack of opportunities for the development of leadership and construction of social skills. This result highlights a very low number of developmental assets in young people, making it necessary to construct strategies that would enable health promotion, which could be based on the expansion of developmental assets (Macêdo and Kublikowski, 2009). These strategies can be envisaged from the results found by Dutra-Thomé et al. (2019) which revealed that high levels of connection with family, community and educational institution were associated with higher rates of self-esteem and self-efficacy and lower levels of involvement in antisocial behavior. These two studies demonstrate that, even in a hostile and vulnerable scenario (Macêdo and Kublikowski, 2009; Instituto Brasileiro de Geografia e Estatística., 2018), as is often the case in developing countries such as Brazil, producing strategies that promote a sense of belonging, support and trust among family members, community and/or educational institution are potentialities that actively contribute to positive youth development (Bowers et al., 2010; Lee and Suzanne-Horsley, 2017; Dutra-Thomé et al., 2019).

Regarding the mental health of young people, studies have confirmed that the development of accepting and mutually beneficial relationships, as well as the construction of a sense of participation and contribution, have been effective in the feeling of psychological well-being when faced with difficulties, in promoting health, and in predicting mental disorders (Fonte et al., 2017; Schotanus-Dijkstra et al., 2017; Milistetd et al., 2021). Longitudinal data have indicated that psychological well-being prevents the first incidence of mental disorders, as well as playing a protective role in the development of any DSM-IV disorder (Schotanus-Dijkstra et al., 2017). Age also seems to contribute to higher levels of positive mental health, as younger individuals had higher rates of positive mental health (Fonte et al., 2017; Milistetd et al., 2021). A hypothesis for these findings was raised by Milistetd et al. (2021), in a study with young Brazilian athletes (e.g., swimmers, judokas, tennis players, basketball players, etc.), which indicated that this phenomenon may be related to the lower level of pressure that the younger athletes (10–13 years of age) experienced, as they are in different developmental stages, with less training, less competitive demands and lower performance expectations. These results were corroborated by Fonte et al. (2017) who highlighted that younger individuals (18–25 years of age) have better mental health and well-being, when compared to older individuals (26–30 years of age).

Promoting psychological well-being factors (e.g., purpose in life, self-acceptance, personal growth) and social well-being (e.g., social acceptance, social contribution) can be a public health strategy to prevent mental disorders such as anxiety, depression, etc. (Schotanus-Dijkstra et al., 2017). These factors are found through the social interactions that occur in all contexts (e.g., family, community, school, etc.) in which young people are inserted. Consequently, it is this combined influence that must be considered when thinking about youth development (Benson et al., 1999; Lerner, 2005; Milistetd et al., 2021). In the case of young Brazilians who play sports, regardless of the type of sport they play, psychosocial development and mental health have more to do with the promotion of quality sporting experiences provided by people who care for the players as a whole and not just as athletes (Milistetd et al., 2021). In addition to sport, the development and maintenance of this type of experience through social skills (e.g., asking for help, expressing feelings, listening empathically, initiating and maintaining conversations, eye contact, intonation and volume of voice, etc.) is a possibility to help individuals deal with various problems, as well as to constructing a support and protection network, especially in cases where they cannot deal with their problems alone (Pereira et al., 2016).

Stressful events also affect young individuals and they can generate adverse outcomes during youth development (Kristensen et al., 2004; Schneider and Pacheco, 2010). They overload individuals and make it difficult to them to develop adaptive functions to deal with this adversity (Kristensen et al., 2010). Exposure to stressful events (e.g., sexual abuse, violence, hunger, unemployment, etc.) can make it difficult for young people to deal with the social demands and expectations placed on them, what affects their mental health. For example, job loss is a stressful event associated with higher levels of psychopathology (e.g., depression, anxiety, stress, etc.; Kristensen et al., 2010; Fonte et al., 2017). When compared to boys, adolescent girls showed higher levels of impact perception and a higher frequency of stressful events related to interpersonal relationships (Kristensen et al., 2010). In another study that investigated the frequency of these events, adolescents considered relatively infrequent stressful events, such as situations of deprivation or loss of freedom and sexual violence, to be of great impact (Kristensen et al., 2004). A relationship was also identified between the experience of stressful events (involving physical, verbal and/or sexual aggression) and antisocial behavior, failure to identify their own mistakes and the development of a persistent pattern of negative, hostile, vindictive and defiant actions against the authority figure (Schneider and Pacheco, 2010).

Seeking a more diversified vision of youth, it is necessary to consider the impact that the experience of positive and/or stressful experiences has on the expansion of developmental assets in young people and to measure their impact on development. Furthermore, when measuring this impact, it is possible to question the roles played by young people in their contexts and the interactions that exist between them (Kristensen et al., 2004; Schneider and Pacheco, 2010). It is therefore relevant for this study to understand how contextual, external and internal assets can affect positive youth development in university students.

### The Brazilian university context

A context to foster the potential of Brazilian young people is Higher Education Institutions (HEIs), which have been expanded in the previous 20 years within the country. In 2017, there was a total of 2,448 HEIs, of which 296 were public HEIs and 2,152 were private (Instituto Nacional de Estudos e Pesquisas Educacionais Anísio Teixeira., 2019). This expansion resulting from programs such as ProUni, Fies, and the Quota Policy (Brasil, 2001, 2005, 2012) has radically changed the profile of Brazilian students. In 2017, there were 3,226,249 newcomers to higher education (55.2% female) throughout Brazil, with 166,203 newcomers participating in the quota program, corresponding to 5.2% of the total (Instituto Nacional de Estudos e Pesquisas Educacionais Anísio Teixeira., 2019). The mean age of entry into the distance learning modality was 31.0 years (SD = 9.2), and in the face-to-face modality was 24.1 years (SD = 7.4; Instituto Nacional de Estudos e Pesquisas Educacionais Anísio Teixeira., 2019).

Entering the university context is a process when change significantly emerges for young people and fosters critical transformations in their lives (Osse and Costa, 2011; Ambiel et al., 2016). It requires adaptations from the new students regarding the way they deal with studies, their interpersonal relationships in the environment inside and outside the institution, new aspects in the learning process and the career transition process (Teixeira et al., 2008; Ambiel et al., 2016). Therefore, young people must manifest complex cognitive and emotional assets to manage the new challenges fostered by this context (Padovani et al., 2014). Adaptation to the university environment can represent a source of problem-solving strategies and socio-emotional development, while it can also result in failures in coping, possibly inducing risk-enhancing behaviors (e.g., use of alcohol and other drugs, unprotected sex, etc.) and psychological distress (Osse and Costa, 2011). This reality has been made explicit since the first study commissioned by the Brasil, 1996, in which highlighted more than 40 causes for university dropout (Brasil, 1996). Reports about the intense demand from studies, difficulties in interpersonal relationships – whether with classmates or with their own family members – difficulties in adapting to the academic reality, and moving from their city of origin to another city, among others, end up revealing the challenges faced by Brazilian university students (Osse and Costa, 2011; Silva and Azevedo, 2018).

These challenges may generate psychological distress. Suicide is the second leading cause of death in the world among people aged 15–29 (World Health Organization [WHO]., 2014). Consisting of specific characteristics, and due to being in the transition between adolescence and adulthood, the period of youth often presents a great internal demand, when they have to manage their actual lives and how society perceives young people (Erikson, 1972; Pereira et al., 2018).

Most university students are in the developmental period designated as emerging adulthood (Arnett, 2011), situated between adolescence and adulthood (18–29 years). This period has been highlighted as a particularly fragile transition for the manifestation of psychopathologies. It is characterized by vast instability in various fields of life (love life, work, etc.), and has five fundamental pillars: (1) it constitutes a moment of identity explorations, with emphasis on career and love life; (2) it is characterized as a time of instability, caused by continuous changes in the social and institutional spheres; (3) it has the feeling in between as a subjective marker, since young people no longer perceive themselves as adolescents and are not yet completely independent adults, however, have characteristics of both; (4) young people tend to be more self-focused, as they do not have fully defined long-term commitments (e.g., children and stable work); and (5) it constitutes a vast field of possibilities, as young people tend to be optimistic about the future, even if their current situation is difficult (Arnett, 2005). For emerging adults, becoming an adult is not manifested through traditional roles (e.g., getting married, having children), but by assuming responsibility for their actions, making independent decisions, and having financial independence (Arnett, 2011). This concept is mainly related to the middle and upper class context of industrialized countries, in which young people find great freedom to make choices and set goals, such as careers and relationships (Arnett, 2005, 2011; Dutra-Thomé and Koller, 2014). However, the autonomy to explore possibilities makes this group more susceptible to risk behaviors, such as drug use (Arnett, 2005).

This study is important because the university is an environment of adaptations inside and outside the university walls because young individuals also need to deal with other microsystem’s demands (e.g., family, community ones, etc., Teixeira et al., 2008; Osse and Costa, 2011; Padovani et al., 2014; Ambiel et al., 2016). As youth developmental trajectories are not fixed and can be significantly influenced by contextual factors, plasticity constitutes an essential element for young people to deal with these changes, defined as the potential they have to systematically change their lives. Plasticity has been shown to be an effective engine for change and transformation (Lerner, 2005; Lerner and Lerner, 2011). In addition, most university students are transitioning into adulthood, a period of identity exploration, instability, and future uncertainty (Arnett, 2005, 2011; Dutra-Thomé and Koller, 2014), mainly in context of political e economic crises, such as in Brazil. Thus, to comprehend the relation of contextual and individual resources and Brazilian youth PYD is relevant because it gives us bases to build intervention and public policies.

Given this scenario, the present study aimed to verify associations between the different forms of social connection (family, community and the educational context), positive mental health indicators, perception of stressful events and the five Cs of PYD (Competence, Confidence, Caring, Character and Connection) in university students aged 18–33 enrolled in public and private HEIs in Brazil. The following specific objectives were set: (1) to verify associations between levels of Positive Mental Health and the five Cs; (2) to verify associations between social connection (family, community and the educational context) and the five Cs; and (3) to verify associations between the perception of stressful events and the five Cs.

The hypotheses that guided this study were: (1) high levels of positive mental health in university students would be positively associated with the five Cs; (2) high levels of university connection would be positively associated with the five Cs; (3) high levels of family connection would be positively associated with the five Cs; (4) high levels of community connection would be positively associated with the five Cs; and (5) a low perception of stressful events would be positively associated with the five Cs.

## Material and methods

### Design

This was a correlational, cross-sectional and descriptive study with a quantitative design. Using the PYD-VSF scale, a preliminary assessment was carried out regarding factors of the five Cs in young Brazilian university students. This study was approved by the ethics committee of the *Instituto de Psicologia* of UFBA under authorization number 3.025.696. It complies with the guidelines of Resolution 196/96 of the National Health Council, as well as the Ethical Criteria for Research with Human Subjects (Resolutions No. 510/2016 and No. 466/2012). Participants who decided to participate in the study signed the consent form. The nature of the study, the confidential nature of the information shared and the data analysis process were explained to the participants.

### Participants

The study sample included 495 university students (74.5% female, 90.3% single). The following inclusion criteria were used: being duly enrolled in a public or private HEI in Brazil and being between 18 and 33 years of age. Participants had different socioeconomic characteristics, were aged between 18 and 33 years (*M* = 23.36 years, SD = 3.59), and self-designated as white (43.2%), brown (29.7%), black (24.6%), Asian (1.8%) and indigenous (0.6%). About 51.1% of the sample lived in households with 3–4 people. In regard to father/caregiver and mother/caregiver, most had technical training (22.2 and 26.3%, respectively) and shared the residence with the participants (Father = 40.8% and Mother 63.4%).

Most of the students (53.3%) received or the family received some type of grant or aid (e.g., food grant, family welfare, emergency aid, scholarship, etc.). Of these, 41.6% claimed or had a family member that claimed the emergency aid proposed by the Brazilian Government, which aimed to provide emergency protection during the crisis period caused by the Coronavirus – COVID-19 pandemic. More than half of the participants were attending public HEIs (77.4%) and were studying in the morning (63.8%). About 51.7% of these students did not participate in activities within or outside the academic environment (e.g., academic directory, religious groups or movements, artistic activities, political groups or movements, volunteer work, and sports teams). The students were distributed in all Brazilian regions, with 60.2% from the Northeast, 22.0% from the Southeast, 13.3% from the South, 2.4% from the Central-West and 2.0% from the North.

Sociodemographic questionnaire, which investigated variables such as: age, city/state of residence, neighborhood of residence, gender, color, parents’ education, property type, marital status, city of origin, and others sharing the household.

Brazilian Youth Questionnaire (*Questionário da Juventude Brasileira*; Phase II Version –Dell’Aglio et al., 2011), from which the following scales were extracted:

#### Connection with the family

The perception of the family relationship is investigated through 15 items, evaluated on a five-point Likert-type scale ranging from (1) “I disagree completely” to (5) “I agree completely.” Examples of items on this scale are: “We are used to talking about problems in our family,” “My parents rarely criticize me” and “I rarely get into fights in my family.” These items have a good degree of reliability, Cronbach’s alpha of 0.86.

#### Connection with the educational institution

The perception of the participants’ relationship with the educational institution is investigated through 7 items, evaluated on a five-point Likert-type scale ranging from (1) “I disagree completely” to (5) “I agree completely.” Examples of items on this scale are: “I feel good when I’m at college/university,” “I like going to college/university” and “I like most of my professors.” These items have a good degree of reliability, Cronbach’s alpha of 0.73.

#### Connection with the community

The perception of the participants’ relationship with the community is investigated through six items, evaluated on a five-point Likert-type scale ranging from (1) “I disagree completely” to (5) “I agree completely.” Examples of items on this scale are: “I can trust people in my community,” “I feel safe in my community” and “I can count on my neighbors when I need them.” These items have a good degree of reliability, Cronbach’s alpha of 0.73.

#### Mental Health Continuum – Short Form

This is a self-report instrument for the assessment of positive mental health, based on symptoms of positive affect, self-development and social connection (see Annex C), and has been validated in Brazil (Machado and Bandeira, 2015). It consists of 14 items answered on a 6-point Likert-type scale, from (1) “never” to (6) “every day.” Examples of items on this scale are: “During the past month, how often did you feel – that people, in general, are good,” “–that you had something important to contribute to society” and “–that you had experiences that challenged you to grow and become a better person.” The Instrument includes three subscales that seek to assess subjectivity or emotional well-being (3 items), psychological well-being (6 items) and social well-being (5 items). This instrument has a good degree of reliability, with Cronbach’s alpha ranging from 0.61 to 0.79. For the structural equations analysis, the total factor of the Mental Health Continuum – Short Form was used, since this score can assess the mental health construct in a broad way. This, in addition to improving the statistical indices of the structural equations analysis, also seemed more appropriate, from the point of view of the authors, as the proposal was to assess mental health as a whole. Furthermore, the items that make up the scale allow its analysis as a general factor.

#### Positive youth development scale – Very Short Form

The PYD – Very Short Form (PYD-VSF) questionnaire, currently being validated in Brazil (Jesus et al., 2021), is a self-report instrument consisting of 17 items to be answered on a 5-point Likert-type scale, ranging from (1) “strongly disagree” to (5) “strongly agree.” The instrument includes 5 subscales, each scale seeking to access one of the five Cs: Competence (3 items, e.g., “I have many friends” and “I do well in my classes”), Confidence (3 items, e.g., “I am happy with myself most of the time” and “I really like the way I look”), Character (4 items, e.g., “I almost never do things that I know I shouldn’t do” and “I enjoy being with people who are of a different race/ethnicity/culture than mine”), Caring (3 items, e.g., “When I see a person taking advantage of another, I want to intervene” and “When I see a person being bullied, I feel sorry for them”) and Connection (4 items, e.g., “I get a lot of encouragement at my college/university” and “I feel like my friends are good friends”). This instrument has a good degree of reliability, with Cronbach’s alphas ranging from 0.37 to 0.90. Due to not presenting a satisfactory factor loading (in this case, 0.187) when the Confirmatory Factor Analyses were performed in factor 3, the item “I almost never do things I know I shouldn’t do” was excluded from the final factor structure of this study. In this study, the PYD – VSF scale with 17 items was used, as it is able to assess the construct. The 34-item version (PYD – SF) needs further studies, therefore, it was not used in this work.

#### Inventory of stressful events (*Inventário de Eventos Estressores* – IEEA)

The IEEA (Ferlin et al., 2000) for adolescents aims to assess the frequency and impact of stressful events (see Annex E). This instrument includes 64 items that assess a range of stressful events that refer to different situations and contexts experienced by young people. For this study, a reduced version of the IEEA was used, containing 21 items. Since the IEEA was initially developed for adolescents, a reduction in the number of items (*n* = 64 to *n* = 21) was carried out so that they were more directed toward the university context and the experience of young people in this environment.

In each of the items, firstly, the subject must indicate whether or not they have experienced the event in their life, indicating “yes” if they have already experienced it and “no” for situations they have never experienced. Immediately afterward, they should indicate, only for the events that they have already experienced, the perceived impact on a five-point Likert-type scale, ranging from “(1) Not at all stressful” to “(5) Extremely stressful.” Examples of items on this scale are: “Having problems with the police,” “Having slept on the street” and “Having difficulties making friends.” In this study, only the indication of having experienced the stressful event or not was used.

### Procedure

In view of the pandemic scenario, caused by the new COVID-19 virus, the application of the instrument was carried out virtually, using an online version of the questionnaire sent to the students of the HEIs on social networks (Instagram, Facebook, etc.) and by email. Before the beginning of the collection, a Pilot Test was carried out with 20 young people, which allowed the level of understanding and the adequacy of the proposed questions to be observed, leading to some reformulations and the exclusion of some items.

### Analysis

Sociodemographic variables were used to characterize the sample through descriptive analysis (e.g., frequency and means). This data can be found on Table 1.

**TABLE 1:** Sociodemographic characteristics: Sex, marital status, age, residential status and who they lived with (*N* = 495).

	*n*	%		*n*	%
Gender			Living in*		
Male	126	25.5	Own property	324	65.5
Female	369	74.5	Rented property University Residence Hostel Other**	140 19 1 9	28.3 3.8 0.2 1.8
Marital status			Living with		
Single	447	90.3	Father	202	40.8
Married	21	4.2	Mother	314	63.4
Cohabiting	22	4.4	Stepfather	24	4.8
Divorced/Separated	4	0.8	Stepmother	4	0.8
			Siblings	205	41.4
Other***	1	0.2	Grandfather Grandmother Uncle/aunt Adoptive parents Children Companion Alone Friends/colleagues Others****	12 46 22 2 12 51 40 47 13	2.4 9.3 4.4 0.4 2.4 10.3 8.1 9.5 2.6
Age (mean, SD)	23.36	3.59			

*493 responses; **Property assigned (borrowed) or invasion; ***No specific designation; ****Cousins, niece, brother-in-law, mother-in-law.

**FIGURE 1:**
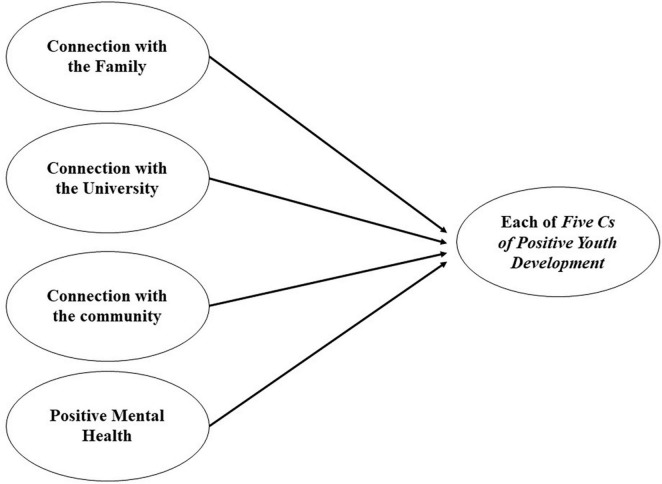
Structural equation analysis model.

Subsequently, Pearson’s correlations were conducted between the five Cs and connection with the family, community, and educational institution; positive mental health; and stressful events. The model was then evaluated through Structural Equation analysis with the five Cs of PYD as dependent variables; and connection with the family, community and educational institution; and positive mental health as independent variables (Figure 1). Comparative Fit Index (CFI), the Tucker-Lewis Index (TLI) and the Root Mean Square Error of Approximation (RMSEA) were calculated as the fit indices of the Structural Equations Model. Following this, standardized regression weights were evaluated to verify associations between the variables. For fit indices, the CFI, TLI, and the RMSEA were calculated with a Confidence Interval (CI) of 90%. Comparative Fit Index and Tucker-Lewis Index values of 0.90 or greater represent an acceptable fit; greater than 0.095 represent a good fit. Root Mean Square Error of Approximation values of 0.08 or less represent an acceptable fit, while values less than 0.05 represent a good fit (Hu and Bentler, 1999). Finally, the sum of the IEAA variables was performed and Pearson’s correlations between the five Cs and stressful events were calculated (see Table 2).

**TABLE 2:** Results of confirmatory factor analysis.

	Five Cs of positive youth development				
	
	Connection	Character	Caring	Confidence	Competence
Connection with the family	0.027	−0.239	−0.344**	0.090*	−0.150
Connection with the university	0.293**	0.136	0.107	0.081*	0.349**
Connection with the community	0.268**	0.053	0.088	0.081*	0.185*
Positive mental health	0.148*	−0.116	−0.157*	0.869**	0.327**
	CFI	TLI	RMSEA	Chi-Square	WRMR
Model FIT	**0.915**	0.908	**0.052 (0.050–0.054)**	**24697.098 (DF 1596)**	**1.524**

Numbers highlighted in bold show significant correlations between the dependent variables (i.e., five Cs of PYD: Connection, Character, Caring, Competence and Confidence) and the independent variables (e.g., perception of the relationship with the family, perception of the relationship with the university, perception of the relationship with the community and positive mental health). CFI, comparative fit index; TLI, Tucker-Lewis index; RMSEA, root mean square error of approximation; WRMR, weighted root mean square residual. *p < 0.05, **p < 0.001.

## Results

Pearson’s correlations with the latent factors of the scales are presented in Table 3. Overall, high levels of positive mental health, connection with the university/college, and connection with the community were positively associated with the Cs of PYD. Competence, Confidence and Connection showed positive and statistically significant correlations with all the independent variables (e.g., connection with the family, connection with the community, connection with the educational institution, and positive mental health). Character showed a positive and statistically significant relationship with all independent variables, except for connection with the community (*r* = 0.088). While Caring showed a positive and statistically significant relationship with connection with the university (*r* = 0.093). There was no statistically significant relationship between Caring and Positive Mental Health (*r* = 0.043), Connection with the community (*r* = 0.074) or Connection with the family (*r* = 0.005).

**TABLE 3:** Correlations between variables.

	Five Cs of positive youth development				
	
	Connection	Character	Caring	Confidence	Competence
Connection with the family	0.526**	0.180**	0.005	0.350**	0.308**
Connection with the university	0.440**	0.172**	0.093*	0.333**	0.361**
Connection with the community	0.449**	0.088	0.074	0.333**	0.313**
Positive mental health	0.533**	0.168**	0.043	0.706**	0.436**

Numbers highlighted in bold show significant correlations between the dependent variables (i.e., five Cs of PYD: Connection, Character, Caring, Competence and Confidence) and the independent variables (e.g., perception of the relationship with the family, perception of the relationship with the university, perception of the relationship with the community, and positive mental health). *p < 0.05, **p < 0.001.

In this study, for analysis purposes, each C of PYD was correlated with the other measures separately. As this is the first study using this construct in Brazil, the proposal was to measure the latent potential of each factor of PYD within the Brazilian context.

Table 2 presents the results of the structural equation analysis having the five Cs of PYD as the dependent variables; and connection with the family, with the community and with the educational institution; and positive mental health as the independent variables and the model fit for the structural equation analysis. The results obtained in the structural equation analysis indicated statistically significant associations, with regression weights that ranged from acceptable to good. Competency had positive and statistically significant relationships with positive mental health (regression weight = 0.327; *p* < 0.000), connection with the university (regression weight = 0.349; *p* < 0.000) and connection with the community (regression weight = 0.642; *p* < 0.014). Confidence was positively and significantly associated with positive mental health (regression weight = 0.869; *p* < 0.000) and with the connection with the university (regression weight = 0.081; *p* < 0.016). Caring had statistically significant negative associations with positive mental health (regression weight = −0.157; *p* < 0.019) and with connection with the family (regression weight = −0.344; *p* < 0.000). Connection showed positive and statistically significant associations with positive mental health (regression weight = 0.148; *p* < 0.005), connection with the university (regression weight = 0.293; *p* < 0.000) and connection with the community (regression weight = 0.268; *p* < 0.000). Finally, Character was not associated with any variable.

Table 4 presents the Pearson’s correlations between the latent factors of PYD and the IEAA. As hypothesized, in general the five Cs of PYD were negatively correlated with the experience of stressful events by university students. However, Caring had a positive and statistically significant correlation with the experience of stressful events (*r* = *0.132*). Therefore, 3 of our hypotheses were confirmed, while one hypothesis was partially confirmed, since Caring had a positive correlation with the experience of stressful events. This result reminds us of the discovery by Geldholf et al. (2019), that developmental regulation can benefit the context at the expense of the individual. Therefore, it is possible for young university students to develop a sense of sympathy and empathy with others and their context at the expense of their own well-being, making the frequency of stressful events higher in this group.

**TABLE 4:** Correlations between the five Cs and stressful events (*N* = 495).

	Competence	Confidence	Character	Caring	Connection
Experience of a stressful event	−0.171**	−0.278**	−0.068	0.132**	−0.264**

**Significant correlation at the 0.01 level.

## Discussion

From the perspective of PYD, the ecological systems experienced by young people directly impact youth development (Dutra-Thomé et al., 2019). Results of the present study showed that high levels of Connection, Confidence, and Competence were associated with high levels of positive mental health in young university students, indicating the importance of symptoms of positive affects, self-development and social connection for positive youth development (Seginer, 2008). This partially confirms the hypothesis that high levels of positive mental health in university students are positively associated with the five Cs. Furthermore, high levels of connection with the university were associated with high levels of Connection, Confidence, and Competence, in part confirming the hypothesis that high levels of connection with the university would be positively associated with the five Cs, as Character and Caring did not demonstrate associations. These results indicate the effectiveness of the connection with the university in promoting factors of protection and promotion for facing adversity, in a context marked by so many challenges (Amparo et al., 2008; Osse and Costa, 2011; Pereira et al., 2016; Telzer et al., 2018). This shows that, in addition to the family and community environments, the university context can present itself as a fundamental catalyst in the development of problem-solving strategies and socio-emotional development (Osse and Costa, 2011). Furthermore, for university students, higher levels of connection with the family were associated with higher levels of Confidence. Here, the internal sense of self-dignity and self-efficacy are strengthened through family connections, which shows the significant the role of the family for youth to follow a life trajectory marked by more beneficial individual-context relationships (Bowers et al., 2010; Crocetti et al., 2014; Årdal et al., 2018). However, this result partially confirms our hypothesis that high levels of family connection would be positively associated with the five Cs, as only Confidence presented a positive association.

Higher levels of connection with the community were associated with higher levels of Connection, Confidence and Competence. Reinforcing the result of Sorhaindo et al. (2016) on the importance of social acceptance in the development of activities and coping with risk-enhancing behaviors. This result partly confirms the hypothesis that high levels of connection with the community would be positively associated with the five Cs. In this case, Lerner et al. (2005) indicated that groups and/or programs that ensure that young people have a sustained relationship with at least one committed adult, who provides opportunities for skills development and works to improve the community, played a key role in the presence of the five Cs for youth development. However, it is necessary that the young person believes in this possibility and invests in this relationship, as this action shows additional advances in the individual development of each young person and also in the health of their social world (Lerner, 2005; Lerner et al., 2005), therefore being able to raise the levels of Caring and Character. Milistetd et al. (2021) also reinforced that carrying out activities and/or sports as a team can reduce disorders that impact the mental health of individuals. However, it is necessary to reiterate the bidirectionality of these social relationships since human development occurs together with the functions and characteristics of the person in the interaction with their environment (Moen et al., 1995). An example of the bidirectionality of the social relationship is the negative association that positive mental health and the perception of the family relationship had with Caring. This result seems to indicate that, for the university students in the sample, having a sense of sympathy and empathy is not positively associated with a sense of well-being, or with the perception of a healthy relationship with the family. When discussing how Caring is not always related to positive youth development, Geldholf et al. (2015) speculated that the positive association caring has with maladaptive outcomes and negative affects could indicate anxiogenic factors and/or emotional hypersensitivity in the young people. Accordingly, as the results of the present study highlight, young people with higher levels of Caring may have a tendency for emotional instability, or there may be a strong tendency to manifest greater anxiety and depressive feelings (Holsen et al., 2017).

The PYD indicators are not strictly one-dimensional. The individual measurements of the five Cs can be correlated with positive and negative outcome variables (Årbeit et al., 2014; Holsen et al., 2017; Geldholf et al., 2019). Each C carries the latent potential to promote maladaptive developmental outcomes such as anxiety, depression, and aggressive behavior. Therefore, the five Cs construct is part of a larger and complex development system that must be seen as multi-valued and analyzed within each specific context (Geldholf et al., 2015, 2019). This must also be taken into account when investigating the negative association between Caring and the connection with the family. This result seems to indicate that having a supportive and beneficial relationship with the family is associated with low levels of Caring. Faced with this indicator, it is necessary to qualitatively verify the empathy of young university students and their belief that it is important to take care of people and the context around them, if necessary. Årbeit et al. (2014) reiterated this need for investigation, as in their study carried out in the United States, for example, young people who used alcohol and presented aggressive behavior were found to have high levels of Confidence and Competence. For these authors, this type of result should work as part of an investigation into the contextual complexity to which young people are subjected, always reiterating the strength that the context exerts, and the adaptability of the PYD measures.

In addition to encouraging promotion and protection factors, it is necessary to think about exposure to risk factors, such as stressful events. In this study, the experience of stressful events presented negative correlations with the five Cs, for the most part. Therefore, university students who showed higher levels of Competence, Confidence, Character and Connection had had less exposure to stressful events, what’s partially confirmed the study hypothesis that a low perception of stressful events would be positively associated with the PYD factors. However, Caring presented a positive relationship with the experience of stressful events. In this sense, the more exposed to stressful events, the more the university student develops and presents a sense of empathy and sympathy. This seems to indicate, as emphasized by a potential risk for the internalization of problems, provoking anxiety about the needs of others (Geldholf et al., 2019). As part of a complex developmental system, each attribute of a given behavior may not necessarily be linked to positive or negative outcomes (Årbeit et al., 2014). The measure of caring still fails to distinguish between individuals who display a sense of empathy and sympathy as part of adaptive or negative developmental regulations (Geldholf et al., 2019). These behaviors need to be interpreted within their context, as drinking alcohol or having an active sex life, for example, can present normative characteristics and add both positive and negative effects (Årbeit et al., 2014). This type of evidence reiterates the force that the context exerts on young people, and the need to consider each society within its practices and culture.

### Final considerations

Results of this study indicated that Brazilian university students present a fragile mental health, especially in what involves the c of care. Despite showing good indicators of connection with others (Connection), of competences and social skills (Competence), of management and knowledge of norms and laws (Character), of confidence in themselves in their attributes (Confidence), participants had their psychological well-being compromised when it comes to being empathetic with others – since results indicated that the c of Care was associated with low levels of psychological well-being. Furthermore, the fact that young people have experienced stressful events at some point in their lives increased their sense of care, characterized by sympathy and empathy toward others. Thus, while experiencing stressful events seems to promote higher levels of care, feeling too much for the other has harmed youth psychological well-being. These results reinforce that the PYD factors need to be seen bidirectionally, that is, they can lead to both satisfactory/healthy and harmful directions for the mental health of young Brazilian university students. For this reason, public policies and interventions aimed at promoting PYD in young people must consider the multiplicity of directions that each of the 5Cs can take, in order to measure the positive and negative impacts they may have.

This article stands out in the study of PYD in Brazil, a country where the measures of the five Cs remained unpublished until now. This preliminary assessment using the PYD-VSF allowed us to identify factors of the five Cs in young Brazilian university students. The results found revealed particularities in the Brazilian context regarding Caring and Character. These initial findings direct us to a research panorama, in which the perspective of PYD becomes available for use in interventions in Brazil.

Limitations include the Coronavirus – COVID-19 pandemic, which caused the questionnaire to be applied virtually, excluding from the sample those students who did not have the internet or had difficulties accessing it. Furthermore, the IEEA may not be sensitive enough to identify the magnitude of experiencing stressful events and/or exposure to risk-enhancing behaviors. This is because the calculation of the sum used in the analysis was not sufficient to verify associations in the structural equation model. In this study, only the external assets of the support category were contemplated through scales that evoke characteristics of the scale developed by the Search Institute. Future studies may consider all the internal and external assets identified by Benson et al. (1999). Finally, the development of a mixed method study could be effective both in measuring the construct and in describing the presence of each aspect of PYD in the lives of these individuals, since Caring presented maladaptive developmental results. Deepening this result may help to clarify whether, in fact, young university students have hypersensitivity to the demands and stressful experiences or whether there is any fragility in the composition of the items in the Caring Factor.

## Data availability statement

The raw data supporting the conclusions of this article will be made available by the authors, without undue reservation.

## Ethics statement

The studies involving human participants were reviewed and approved by Comitê de Ética do Instituto de Psicologia da UFBA. The patients/participants provided their written informed consent to participate in this study.

## Author contributions

MJ and AP organized the database and performed the statistical analysis. MJ wrote the first draft of the manuscript. MJ and LD-T contributed to the conception and design of the study, wrote sections of the manuscript, and manuscript revision, read, and approved the submitted version.

## References

[B1] Adams B. G. Wiium N. Abubakar A. (2019). Developmental assets and academic performance of adolescents in Ghana, Kenya S. Africa. *Child Youth Care Forum* 48 207–222. 10.1007/s10566-018-9480-z

[B2] Ambiel R. A. M. Dos Santos A. A. A. Dalbosco S. N. P. (2016). Motivos para evasão, vivências acadêmicas e adaptabilidade de carreira em universitários. *Psico* 47:288. 10.15448/1980-8623.2016.4.23872

[B3] Amparo D. M. do Galvão A. C. T. Alves P. B. Brasil K. T. Koller S. H. (2008). Adolescentes e jovens em situação de risco psicossocial: Redes de apoio social e fatores pessoais de proteção. *Estudos de Psicologia (Natal)* 13 165–174. 10.1590/S1413-294X2008000200009

[B4] Årbeit M. R. Johnson S. K. Champine R. B. Greenman K. N. Lerner J. V. Lerner R. M. (2014). Profiles of problematic behaviors across adolescence: Covariations with indicators of positive youth development. *J. Youth Adolesc.* 43 971–990. 10.1007/s10964-014-0092-0 24562425

[B5] Årdal E. Holsen I. Diseth Årdal Larsen T. (2018). The five CS of positive youth development in a school context; gender and mediator effects. *Sch. Psychol. Int.* 39, 3–21. 10.1177/0143034317734416

[B6] Arnett J. J. (2005). The developmental context of substance use in emerging adulthood. *J. Drug Issues* 35 235–254. 10.1177/002204260503500202

[B7] Arnett J. J. (2011). “Emerging adulthood(s): The cultural psychology of a new life stage,” in *Bridging cultural and developmental approaches to psychology: New syntheses in theory, research, and policy*, ed. Jensen L. A. (Oxford), 255–275. 10.1007/s11524-013-9827-6

[B8] Benson P. L. Scales P. C. Leffert N. Roehlkepartain E. C. (1999). *A fragile foundation: The state of developmental assets among American youth.* Minneapolis, Minn: Search Institute.

[B9] Bowers E. P. Li Y. Kiely M. K. Brittian A. Lerner J. V. Lerner R. M. (2010). The five Cs model of positive youth development: A longitudinal analysis of confirmatory factor structure and measurement invariance. *J. Youth Adolesc.* 39 720–735. 10.1007/s10964-010-9530-9 20397040

[B10] Brasil (1996). Mistério da educação e cultura. Secretaria da educação superior. *Comissão especial de estudos sobre a evasão nas universidades públicas brasileiras.* Brasília: ANDIFES/ABRUEM/SESU/MEC.

[B11] Brasil (2001). *Lei n. 10.260: Dispõe sobre o fundo de financiamento ao estudante do ensino superior e dá outras providências. Brasília: Diário Oficial da União*, 2.

[B12] Brasil (2005). *Lei n. 11.096: Institui o programa universidade para todos–prouni, regula a atuação de entidades beneficentes de assistência social no ensino superior, altera a Lei n 10.891, de 9 de julho de 2004, e dá outras providências. Brasília: Diário Oficial da União*, 7.

[B13] Brasil (2012). *Lei n. 12.711: Dispõe sobre o ingresso nas universidades federais e nas instituições federais de ensino técnico de nível médio e dá outras providências. Brasília: Diário Oficial da União*, 1.

[B14] Bronfenbrenner U. (1998). *A ecologia do desenvolvimento humano: Experimentos naturais e planejados.* Porto Alegre: Artes Médicas.

[B15] Copetti F. Krebs R. J. (2004). “As propriedades da pessoa na perspectiva do paradigma bioecológico,” in *Ecologia do desenvolvimento humano: Pesquisa e intervenção no Brasil*, ed. Koller S. H. (São Paulo: Casa do Psicólogo), 67–89.

[B16] Crocetti E. Erentaitė R. Žukauskienė R. (2014). Identity styles, positive youth development, and civic engagement in adolescence. *J. Youth Adolesc.* 43 1818–1828. 10.1007/s10964-014-0100-4 24488126

[B17] Dell’Aglio D. D. Koller S. H. Cerqueira-Santos E. Colaço V. (2011). “Revisando o questionário da juventude brasileira: Uma nova proposta,” in *Adolescência e juventude: Vulnerabilidade e contextos de proteção*, eds Dell’Aglio D. D. Koller S. H. (São Paulo: Casa do Psicólogo), 259–270.

[B18] Dutra-Thomé L. Koller S. H. (2014). Emerging adulthood in brazilians of differing socioeconomic status: Transition to adulthood1. *Paidéia (Ribeirão Preto)* 24 313–322. 10.1590/1982-43272459201405

[B19] Dutra-Thomé L. DeSousa D. Koller S. H. (2019). Promotive and risk factors for positive youth development among emerging adults in Brazil. *Child Youth Care Forum* 48 171–185. 10.1007/s10566-018-9475-9

[B20] Erikson E. H. (1972). *Identidade, juventude e crise.* Rio de Janeiro: Zahar.

[B21] Ferlin M. Lima J. S. Alchieri J. C. Kristensen C. H. Flores R. Z. (2000). *Desenvolvimento do inventário de eventos estressores na adolescência (IEEA). Resumos das Comunicações – Exponha-se – Semana de Pesquisa e Iniciação Científica.* São Leopoldo: UNISINOS, 204–205.

[B22] Fonte C. Ferreira C. Alves S. (2017). Estudo da saúde mental positiva em jovens adultos: Relações entre psicopatologia e bem-estar. *Rev. Psique* XIII, 57–74.

[B23] Geldholf G. J. Bowers E. P. Mueller M. K. Napolitano C. M. Callina K. S. Walsh K. J. (2015). *The five Cs model of positive youth development. In Promoting positive youth development: Lessons from the 4-H study.* Cham: Springer International Publishing, 161–186. 10.1007/978-3-319-17166-1_9

[B24] Geldholf G. J. Larsen T. Urke H. Holsen I. Lewis H. Tyler C. P. (2019). Indicators of positive youth development can be maladaptive: The example case of caring. *J. Adolesc.* 71 1–9. 10.1016/j.adolescence.2018.11.008 30583200

[B25] Holsen I. Geldhof J. Larsen T. Aardal E. (2017). The five Cs of positive youth development in Norway: Assessment and associations with positive and negative outcomes. *Int. J. Behav. Dev.* 41 559–569. 10.1177/0165025416645668

[B26] Hu L. Bentler P. M. (1999). Cutoff criteria for fit indexes in covariance structure analysis: Conventional criteria versus new alternatives. *Struct. Equ. Modeling Multidiscip. J.* 6 1–55.

[B27] Instituto Brasileiro de Geografia e Estatística. (2018). *Pesquisa nacional por amostra de domicílios contínua - PNAD (3a ed.).* Rio de Janeiro. *c.

[B28] Instituto Nacional de Estudos e Pesquisas Educacionais Anísio Teixeira. (2019). *Resumo técnico do censo da educação superior 2017.* Brasília: MEC.

[B29] Jesus M. C. Pereira A. S. Dutra-Thomé L. (2021). *Validação da escala de desenvolvimento juvenil positivo–short form [Manuscript not published].*

[B30] Kozina A. Wiium N. Gonzalez J.-M. Dimitrova R. (2019). Positive youth development and academic achievement in slovenia. *Child Youth Care Forum* 48 223–240. 10.1007/s10566-018-9457-y

[B31] Kristensen C. H. Leon J. S. D’Incao D. B. Dell’Aglio D. D. (2004). Análise da frequência e do impacto de eventos estressores em uma amostra de adolescentes. *Interação em Psicol.* 8 45–55. 10.5380/psi.v8i1.3238

[B32] Kristensen C. H. Schaefer L. S. Busnello F. de B. (2010). Estratégias de coping e sintomas de stress na adolescência. *Estudos de Psicol. (Campinas)* 27 21–30. 10.1590/S0103-166X2010000100003

[B33] Lee A. R. Suzanne-Horsley J. (2017). The role of social media on positive youth development: An analysis of 4-H Facebook page and 4-H’ers’ positive development. *Child. Youth Serv. Rev.* 77 127–138. 10.1016/j.childyouth.2017.04.014

[B34] Lerner R. M. (2005). *Promoting positive youth development: Theoretical and empirical bases. (2005). Texto preparado para o workshop on the science of adolescent health and development, national research council/institute of medicine.* Washington, DC: National Academies of Science.

[B35] Lerner R. M. Lerner J. V. (2011). *The positive development of youth: Report of the findings from the first seven years of the 4-H study of positive youth development.* Somerville, MA: Institute for Applied Research in Youth Development.

[B36] Lerner R. M. Lerner J. V. Almerigi J. B. Theokas C. Phelps E. Gestsdottir S. (2005). Positive youth development, participation in community youth development programs, and community contributions of fifth-grade adolescents: Findings from the first wave of the 4-H study of positive youth development. *J. Early Adolesc.* 25 17–71. 10.1177/0272431604272461

[B37] de Macêdo R. M. S. Kublikowski I. (2009). Valores positivos e desenvolvimento do adolescente: Perfil de jovens paulistanos. *Psicol. Em Estudo* 14 689–698. 10.1590/S1413-73722009000400009

[B38] Machado W. de L. Bandeira D. R. (2015). Positive mental health scale: Validation of the mental health continuum–short form. *Psico-USF* 20 259–274. 10.1590/1413-82712015200207

[B39] Milistetd M. Camiré M. Ciampolini V. Quinaud R. T. Nascimento J. V. do. (2021). Psychosocial development and mental health in youth Brazilian club athletes: Examining the effects of age, sport type, and training experience. *Rev. Brasileira de Cineantropometria Desempenho Humano* 23:e78769. 10.1590/1980-0037.2021v23e78769

[B40] Moen P. Elder G. H. Luscher K. (1995). *Examining lives in context: Perspectives on the ecology of human development.* Washington, DC: American Psychological Association, 563–597.

[B41] Osse C. M. C. Costa I. I. (2011). Saúde mental e qualidade de vida na moradia estudantil da Universidade de Brasília. *Estudos de Psicologia (Campinas)* 28 115–122. 10.1590/S0103-166X2011000100012

[B42] Padovani C. Neufeld C. B. Maltoni J. Barbosa L. N. F. de Souza W. F. (2014). Vulnerability and psychological well-being of college student. *Rev. Brasileira de Terapias Cognitivas* 10 02–10. 10.5935/1808-5687.20140002

[B43] Pereira A. S. Dutra-Thomé L. Koller S. H. (2016). Habilidades sociais e fatores de risco e proteção na adultez emergente. *Psico* 47:268. 10.15448/1980-8623.2016.4.23398

[B44] Pereira A. S. Willhelm A. R. Koller S. H. de Almeida R. M. M. (2018). Fatores de risco e proteção para tentativa de suicídio na adultez emergente. *Ciênc. Saúde Colet* 23 3767–3777. 10.1590/1413-812320182311.29112016 30427447

[B45] Schneider A. M. de A. Pacheco J. T. B. (2010). Eventos estressores e conduta social na adolescência. *Gerais Rev. Int. Psicol.* 3 23–32.

[B46] Schotanus-Dijkstra M. Ten Have M. Lamers S. de Graaf R. Bohlmeijer E. T. (2017). The longitudinal relationship between flourishing mental health and incident mood, anxiety and substance use disorders. *Eur. J. Public Health* 27, 563–568. 10.1093/eurpub/ckw202 27818372

[B47] Seginer R. (2008). Future orientation in times of threat and challenge: How resilient adolescents construct their future. *Int. J. Behav. Dev.* 32 272–282. 10.1177/0165025408090970

[B48] Silva M. V. M. da Azevedo A. K. S. (2018). Um olhar sobre o suicídio: Vivências e experiências de estudantes universitários. *Rev. Psicol. Diversidade e Saúde* 7:400. 10.17267/2317-3394rpds.v7i3.1908

[B49] Sorhaindo A. Mitchell K. Fletcher A. Jessiman P. Keogh P. Bonell C. (2016). Young women’s lived experience of participating in a positive youth development programme: The “teens & toddlers” pregnancy prevention intervention. *Health Educ.* 116 356–371. 10.1108/HE-01-2015-0002

[B50] Teixeira M. A. P. Dias A. C. G. Wottrich S. H. Oliveira A. M. (2008). Adaptação à universidade em jovens calouros. *Psicol. Escolare Educac.* 12 185–202. 10.1590/S1413-85572008000100013

[B51] Telzer E. H. van Hoorn J. Rogers C. R. Do K. T. (2018). “Social influence on positive youth development: A developmental neuroscience perspective,” in *Advances in child development and behavior*, ed. Benson J. B. (Cambridge, MA: Elsevier Academic Press), 215–258. 10.1016/bs.acdb.2017.10.003 PMC6345387 29455864

[B52] Tirrell J. M. Geldhof G. J. King P. E. Dowling E. M. Sim A. T. R. Williams K. (2019). Measuring spirituality, hope, and thriving among salvadoran youth: Initial findings from the compassion international study of positive youth development. *Child Youth Care Forum* 48 241–268. 10.1007/s10566-018-9454-1

[B53] Wiium N. Dost-Gözkan A. Kosic M. (2019). Developmental assets among young people in three european contexts: Italy, Norway and Turkey. *Child Youth Care Forum* 48 187–206. 10.1007/s10566-018-9446-1

[B54] World Health Organization [WHO]. (2014). *Preventing suicide: A global imperative.* Available online at: http://apps.who.int/iris/bitstream/10665/131056/1/9789241564779_eng.pd *A

